# “We cannot live like Canadian”: Yazidi refugees’ perspectives on mental health, coping strategies and barriers to care

**DOI:** 10.3389/fpsyt.2025.1623358

**Published:** 2025-10-21

**Authors:** Jacqueline Bobyn, Bethel Abraham, Nicole Kain, Kimberly Williams, Annalee Coakley, Rita Watterson

**Affiliations:** 1 Department of Psychiatry at University of Calgary, Cumming School of Medicine, Calgary, AB, Canada; 2 Department of Global and Environmental Health, New York University School of Global Public Health, New York, NY, United States; 3 Faculty of Medicine and Dentistry, University of Alberta, Edmonton, AB, Canada; 4 Department of Family Medicine at the University of Calgary, Cumming School of Medicine, Calgary, AB, Canada

**Keywords:** Yazidi, culturally sensitive, refugee mental health, culturally informed care, mental health

## Abstract

**Background:**

The Yazidi people are a Kurdish religious minority group who have been persecuted by the Islamic State of Iraq and Syria (ISIS). The complexity of the trauma the Yazidi people endured, and a limited understanding of their illness belief models have created challenges to providing culturally sensitive psychiatric care. The purpose of this study was to use focus group methodology to understand Yazidi refugees’ experiences, to provide culturally informed mental health care.

**Methods:**

Two in-person focus groups were held in Calgary, Alberta with Yazidi refugee women from Iraq and Syria (N = 6, N = 7) to assess perspectives on mental health, preferred coping strategies and perceived barriers to care. Participants were selected using purposive sampling. Focus group design and facilitation were done in partnership with Yazidi cultural brokers and interpreters. Focus groups were conducted in English and interpreted in Kurmanji. The focus groups were recorded, coded, and subjected to qualitative content thematic analyses. The analysis was guided by an interpretivist epistemology and informed by pragmatism, to situate participants’ perspectives within their social context while generating culturally informed insights for psychiatric care in Canada.

**Results:**

Experiences with psychiatric symptoms (e.g. grief and loss, somatization, depression, trauma) were identified. Family reunification and community support were emphasized as preferred coping methods. Perceived unrealistic expectations of refugees post-migration, social isolation and language difficulties were acknowledged as barriers to care.

**Conclusion:**

Providing appropriate psychiatric care to Yazidi refugee women requires a culturally informed approach. Findings in this study support the need for culturally sensitive mental health interventions in refugee populations post migration.

## Introduction

The Yazidi people are a resilient, Kurdish religious minority group that have been severely persecuted. Originating from northern Iraq, western Iran, eastern Turkey and northern Syria, this population has been oppressed over the last 800 years, with estimates of up to 74 genocides carried out against them (1, 2). Since 2014, when the Islamic State of Iraq and Syria (ISIS) announced an Islamic Caliphate in these regions, there are estimates that more than 7,000 Yazidi people have been killed (1, 3). There have also been horrific accounts of systemic violence carried out against the Yazidi people including torture, enslavement, forcible transfer and sexual slavery (1, 2). These recent human rights violations contribute to the longstanding persecution this minority group has faced.

High rates of mental and physical health comorbidities have been documented in the Yazidi population now residing in displacement camps and refugee receiving countries around the world related to their experience of genocide. The prevalence of post-traumatic stress disorder (PTSD) and major depressive disorder (MDD) amongst Yazidi women has been reported as high as 90 percent (1). Furthermore, high rates of psychosomatic symptoms amongst the Yazidi population, including chronic headaches, abdominal, and pelvic pain have been noted (2, 4). Symptoms of functional neurological disorder, such as functional seizures are also acknowledged (2, 5). Although functional seizures may appear like epileptic seizures to non-medical professionals, the etiology of these seizures may be related to an underlying psychological illness such as extreme psychological stress as opposed to epileptic brain activity (2). These mental and physical health comorbidities are thought to impede this population’s ability to function and thrive in displacement camps, and in refugee receiving countries post migration.

As seen in refugee receiving countries around the world, Yazidi patients in Canada are also presenting with mental health concerns. As noted by Hassan et al. (6), between the years 2017–2018 Canada resettled approximately 1,500 Yazidi refugees shortly after rescue. The majority of these were women and children. This relatively short period of resettlement after rescue contrasts with the experience of many refugees who spend years in refugee camps before being resettled (6). Hassan et al. (6) documented the health status of 242 Yazidi refugees seen at a Canadian refugee clinic between 2017 and 2018. Findings revealed that one-third of this population had diagnosed mental health conditions thought to be associated with ISIS exposure, with symptoms such as nightmares, panic attacks, eating disorders and suicidal ideation noted. Hassan et al. (6) also found that five percent of Yazidi women at this clinic struggled with functional neurological symptoms in the form of functional seizures. Functional seizures in this population have been reported as high as forty-four percent in other studies (5). However, population studies in the United States and northern Europe estimate the incidence of persistent functional neurological symptoms as four to twelve per 100,000 per year (7). Therefore, there may be a potentially higher incidence of these symptoms in the Yazidi population based on these findings (6, 8).

Providing culturally sensitive mental health care to address these concerns has been challenging. In Canada, Yazidi refugees’ experiences with accessing health care have included frequent clinic visits, numerous emergency presentations and inpatient psychiatric admissions. Treatment efforts involving medications and psychotherapy such as cognitive behavioral therapy have been trialed with limited success (8).

Providing mental health care to refugees is challenging due to healthcare providers’ lack of knowledge of mental health perspectives within the population, misunderstanding of normative coping methods, and ignorance of the barriers faced by this population to accessing care. Having a limited understanding of the Yazidi people’s illness belief models contributes to the difficult nature of providing holistic care. Furthermore, it may also be hypothesized that having a limited understanding of the Yazidi people’s preferred coping strategies and a lack of awareness of their own perceived barriers to care may also be contributing to the challenges faced by care providers.

In order to adequately address these concerns, further research is needed. Focus groups are particularly helpful as a dynamic and interactive discussion can evoke memories and encourage participants to share insights in ways that they may not be able to with other methods such as through quantitative scales or more structured single interviews (9). This study investigated Yazidi refugee’s perspectives on their mental health, their preferred ways of coping, and their perceived barriers to receiving mental health care in a Canadian health care system. It is part of a larger overarching exploration to providing mental health care to Yazidi refugees, which also included front-line service providers’ experiences of providing care. The aim of this research is to address these evident knowledge gaps and unveil strategies for delivering culturally sensitive mental health care to Yazidi refugees going forward.

### Methods

This study was a collaborative effort between the Department of Psychiatry at the Cumming School of Medicine at the University of Calgary, the Mosaic Refugee Health Clinic, and the Calgary Community and Refugee Alliance Centre all of which are located in Calgary, Alberta, Canada. The Mosaic Refugee Health Clinic (now known as the Calgary Refugee Health Clinic) is a center in Calgary where refugees from around the world receive primary and specialized health care for their first two years after arrival in Canada (10 From a mental health perspective, psychologists, social workers, nurses, family physicians and psychiatrists collaborate to provide mental health support (11, 12). The Calgary Community and Refugee Alliance Centre is a society that promotes the mental health of newcomers by focusing more broadly on the social determinants of health. The Centre’s overarching goal is to connect refugees with community programs and services that most benefit them, while strengthening the communities’ capacity to reach their potential (Calgary Refugee Health Society, n.d.). The study’s protocol was approved by the Conjoint Research Ethics Board at the University of Calgary (Study ID: REB 20-1774).

### Participants

Three focus groups were conducted to describe the meaning and significance of Yazidi refugees experiences within the Canadian mental health care system. Two focus groups consisted of Yazidi refugee women (from Iraq or Syria) who a) re-settled to Canada between the years 2014-2020; b) were admitted to Canada either as a government sponsored or a privately sponsored Yazidi refugee; c) were able to speak Kurmanji or English and d) had been affiliated with the Calgary Community and Refugee Alliance Centre. Exclusion criteria included males (given the fact that the Yazidi culture is very divided by gender roles, and having members of the opposite gender may have risked social desirability bias), anyone under the age of 18 (adult participants only given that this was the cohort of interest) and any individual suffering from symptoms of an unspecified schizophrenia spectrum and other disorder, and substance use disorders, as these symptoms may have interfered with the ability to participate in this setting. As part of the larger overarching study, a third focus group consisted of front-line mental health service providers working at the Mosaic Refugee Health Clinic. This manuscript details the experiences of Yazidi refugees from the first two focus groups; a subsequent publication will describe the learnings from the third focus group.

Yazidi refugee women were selected using purposive sampling at the Bowness Community Association, a location where the Calgary Community and Refugee Alliance Centre held weekly community gatherings. Participants were approached using professional interpreting services. During recruitment it was made clear that participation was voluntary and declining to participate would have no impact on the care received through the Calgary Community and Refugee Alliance Centre. The informed consent process was then carried out in person with interested participants using professional interpreting services. Informed consent was gathered either in written format or verbally (given that the majority of the Yazidi women were illiterate). If the women were illiterate, but they wished to provide their signature, this opportunity was provided. However, if they were unable to sign their name, a witness was available to sign their own name indicating that verbal consent was provided. The first focus group consisted of six Yazidi refugee women and the second focus group consisted of seven Yazidi refugee women. Participants ranged in age between 18 and 60 years.

### Focus group discussions

Focus group questions were created with the help of Yazidi cultural brokers. Specifically, it was important for this study to be done in partnership with Yazidi refugee community leaders. This was done to ensure that the questions posed were culturally sensitive, and validated, prior to the delivery of the focus groups themselves. The same Yazidi cultural brokers were also present during the administration of the focus groups to ensure appropriate cultural context.

Semi-structured focus group discussions with the Yazidi refugee women were conducted in private outdoor park settings. To ensure cultural sensitivity, traditional food and drink were served, and participants were encouraged to bring their children to play in nearby park spaces. Childcare was provided. These focus groups were run with two clinician researchers and two Yazidi cultural brokers. Focus groups were conducted in English and interpreted into Kurmanji, the native Yazidi language, by the Yazidi cultural brokers who were fully trained as interpreters.

At the beginning of each focus group discussion, the research study was again described, and participants were encouraged to participate. Each participant had an opportunity to withdraw their consent just prior to the start of the discussion. Questions pertaining to Yazidi perspectives on mental health, perceived barriers to receiving mental health care and preferred coping strategies were asked. Clarification was provided when needed. Focus group discussions lasted approximately 120 minutes. All focus groups discussions were audio recorded for transcription. Given the sensitive nature of the topics explored, grounding exercises and periodic check-ins with participants were done throughout the focus groups. Facilitated debriefs were also held after completion of the focus groups to ensure participant safety prior to completion of participation.

### Analysis & methodological framework

Audio recorded data was transcribed and coded manually (JB and BA). Themes were identified and quotes from discussions were noted for each theme. Themes that represented the most frequently occurring ideas were reported. Codes, thematic content and data categorization were reviewed by the other authors for quality control. Excel was used to record themes and manually code thematic content.

This study is grounded in an interpretivist epistemology, emphasizing that meaning is contextually situated and co-shaped through human interaction which is critical in culturally and trauma-informed qualitative research (13). Given the objective of informing culturally sensitive psychiatric care, this work also draws on a pragmatic orientation. Pragmatism allows for the use of qualitative content analysis and thematic coding to generate findings that are directly applicable to practice, emphasizing knowledge that is both contextually grounded and action-oriented (14, 15).

## Results

**Figure 1:**
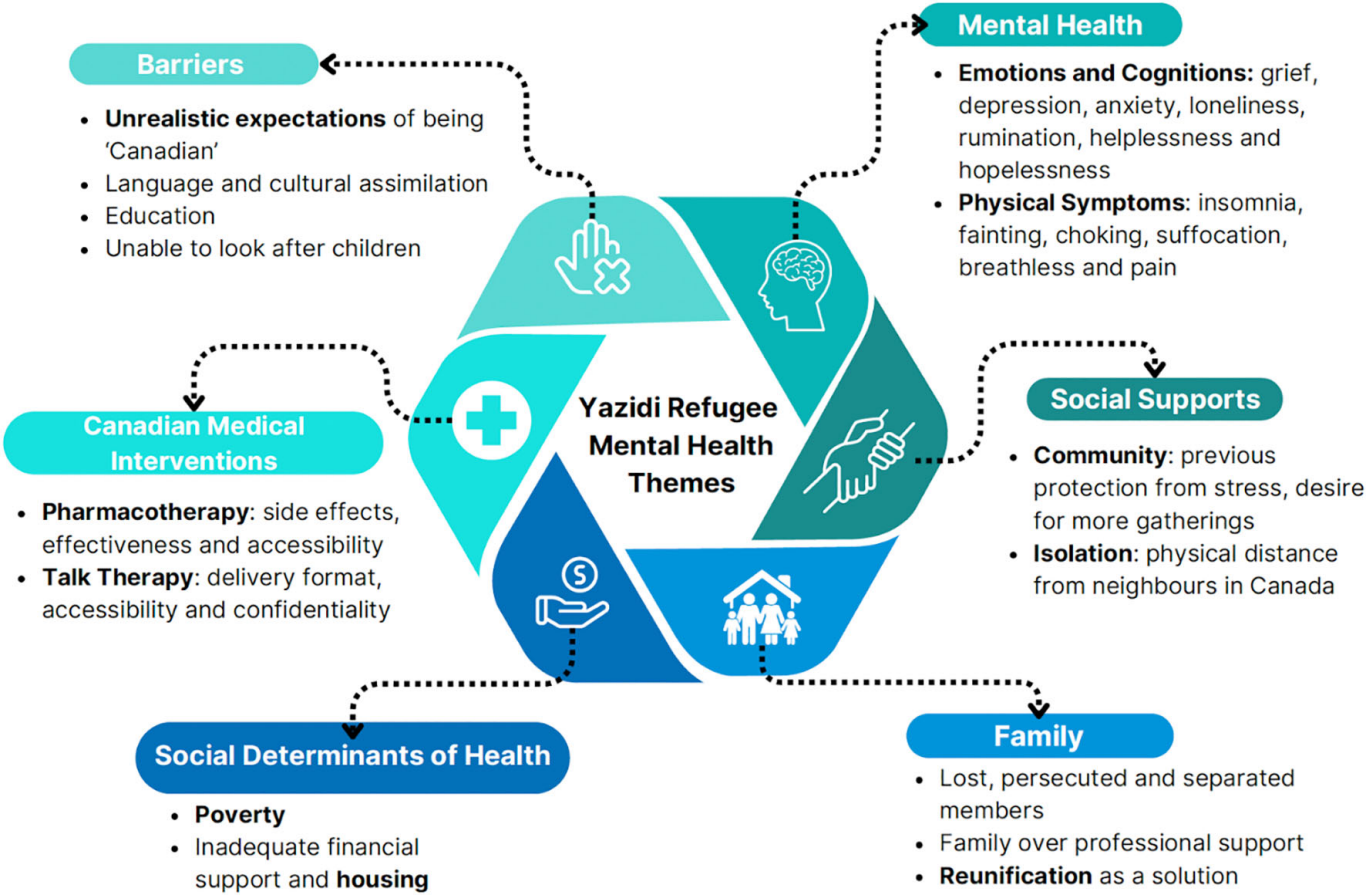
Yazidi refugee mental health themes.

Six themes regarding Yazidi experiences with mental health, their preferred coping mechanisms and barriers to care were identified: Mental Health, Family, Canadian Medical Interventions, Social Supports, Social Determinants of Health and Barriers (see **Figure 1**).

### Mental health

When Yazidi women were asked to describe what stress is, they mainly described the significant trauma they endured, and associated post-traumatic, somatic and depressive symptomatology. For example, sensations of not being able to “breathe”, and experiences of “headaches, insomnia, fatigue and fainting” were frequently noted by several women. One woman summarized this, explaining “everyone with mental problems would have different reactions, some people would faint, some would have headache, some people would have body … um just not feeling well to do anything.” One participant acknowledged “when I feel that my anxiety is increasing … um I get the headache, I just want to walk away, to be alone in a park, my body is tired.”

### Family

All Yazidi participants recognized that lost, missing, and persecuted family members contributed to the main perpetuating cause of their distress. One woman shared “personally, when I remember that my kids are still in captivity … no one can help us with this problem.” It was emphasized that “stress is when we know that our family members are missing, and we know that they suffered a lot and we still don’t know anything about them.” It was also discussed that this is a very common shared experience for many of the women. One participant explained “I am not the only mother that is suffering and going through this. There are so many moms that are going through this. When it comes to kids, it’s on top of everything … if every morning I don’t take a pill for stress and mental problems, I would be crazy now.”

### Canadian medical interventions

When asked how their experiences have been receiving help in Canada, participants emphasized an appreciation for doctors, and universal health care. “They are providing everything for us until now and we really appreciate it … they provide everything for free … we don’t have to pay for these supports” one woman explained. “The medications are always on time. The medications come regularly” another participant agreed. The support of specialized refugee health clinics was also noted as one woman described “in Canada all of the doctors here, especially at the refugee clinic, they were supporting us a lot. But it is not enough for what we went through.” This notion that their problems are not curable by the existing mental health system was felt by many. For example, one woman shared that “no matter how much medication we take … somehow I keep thinking about what happened.” Another participant echoed this sentiment when she shared “because it has been seven years and I don’t know anything about my son, or my daughter, or my husband. When it comes to my mind, not any medications or doctors or anybody will help. I will go crazy.” Others emphasized that “Canada has really helped a lot … we will never forget that, and we appreciate that. But because of what happened we cannot get rid of our stress and mental problems.”

Lastly, a preference for receiving help from family members over practitioners was emphasized. One participant noted that back home when people struggled with mental health “there were family members and family members were more important than doctors.” Another woman agreed, sharing that “when I feel stress, I would feel more … comfortable to talk to my mom, sister or brother. I would trust them more than the doctors. I can open my heart to them.”

### Social supports

Relying on social supports was identified as a preferred coping strategy for many of the women. Many of them emphasized how in Canada this has been difficult to do, however. One woman explained “back home when someone was passed … when we lost someone, we would all get together as a community and for a year, we would always like be in that house that they lost the person … to less[en] the stress, to take care of each other … But here it is different, because here everyone is really far from each other.” Another woman explained that back home “it wasn’t that hard for us because usually the community would help … they would give their harvest to their neighbors. Now the stress is different for us.” Finally, a desire for more opportunities for connection was evident. One woman emphasized “having gatherings with the women like this it would really help … and I know when we go back home, we are again going to have stress … sitting home by ourselves. Once in a while if we do something like this, gather, talk with the ladies it would help.”

### Social determinants of health

The Yazidi women emphasized that inadequate housing and financial pressures perpetuate the ongoing stress that they experience post-migration. For example, one woman explained “even the houses we are in now, so many things need to be fixed and the landlord is just not fixing it. The tub and sink and dryer are not working. I had to use a towel to dry the water that is coming from the machine.” Others expressed concern that current living conditions reminded them of captivity, particularly those residing in basement suites. One woman stated “for stress and mental problems … it is not really a good idea to live in a basement” and that if they could get access to further financial help, then one could get a house that is “upstairs or something.” Lastly, concerns over having to leave their children to obtain money were expressed. For example, one woman explained “I do not have a husband. I have two kids and they are little. They are unable to work. The government is forcing us to work and leave our kids. I do not know the language, and this makes it even more hard.”

### Barriers

Language barriers were identified as an ongoing contributor to isolation. One woman shared “back home even our neighbors we would all speak the same language. But here our neighbors they don’t speak the same language, so we aren’t able to communicate with them. They are either busy with the kids, school, doctor appointments and stuff like this … so we don’t get the chance to talk to anybody.” When speaking of language classes, one woman explained that “I have now been in the same classroom for three years … its not helping me … and I am not improving … my teacher always considers me like a regular student, but it shouldn’t be this way. I have mental problems and health problems. I am not supposed to be considered a regular student … that is what is causing me stress.” Another participant expressed how foreign it was to attend school, something they had never done before. “It is still difficult for four to five hours sitting in the same chair, it is very difficult” she said. This sentiment of unrealistic expectations was shared among many. An older participant explained “People like our age … how are they able to attend school? At this age, going to school and learning. I have diabetes, I have so many medications to take. I have to go to wake up in the morning to go to school, come back, study, take care of my kids, how am I able to do all of this at this age? Doctors need to know this.” Another participant agreed with this explicitly noting “the expectations are too high.”

Finally, cultural differences were also highlighted as an ongoing barrier. Specifically, an inability to honor collectivistic family priorities was noted. One participant elaborated “the government is forcing us to work and leave our kids,” another explained “I have kids but we have no time. They are going to school. We are not spending enough time together.” One participant elaborated that “ if you have kids and need to take time off, as soon as you take time off from school the financial aid stops”. This makes it challenging to honor collectivistic child rearing practices. One woman explained that back home that other women in the community are “helping out … if you have any problems, the ladies are able to stay home … they can stay home and get the food ready for when the kids come home”. Due to refugee financial aid being tied to their English class attendance in Canada, this collectivistic child rearing practice is more difficult. As one woman summarized “I have to support my family. We cannot live like Canadian. Because we have to cook every day for our kids and do everything for them. Our culture is not like Canadian culture.”

## Discussion

This study addresses gaps in the literature by exploring Yazidi refugee’s perspectives on their mental health, their preferred ways of coping, and their perceived barriers to receiving mental health care in a Canadian healthcare setting. Findings identified how mental health challenges are expressed and further perpetuated. Specific Canadian medical interventions and social supports were emphasized as this Yazidi community’s preferred ways of coping. Notably, the universal health care model, along with specialized refugee health clinics, were deemed as helpful Canadian medical interventions. However, despite appreciating this care, some participants felt that their mental health struggles will never go away. Part of this may be a result of these participant’s noted preference for seeking out support from their families and communities, over doctors. The reality that many of these participants have deceased, lost, or missing family members perpetuates this complexity further. Lastly, these groups of Yazidi refugee women also identified several social determinants of health, language differences, social isolation and unrealistic expectations of the Canadian health care system as ongoing barriers to receiving mental health care in Canada.

While discussing perspectives on mental health, participants in this study acknowledged several ways in which mental distress is expressed physically in the body. Difficulties with pain, headaches, perceived sensations of choking, suffocation, and fainting were acknowledged as common symptoms in those with mental health struggles. This is consistent with previous findings in the literature. In their review of medically unexplained physical symptoms in refugees, Rohlof et al. (16) concluded that refugees from non-western countries exhibit more unexplained somatic symptoms than the general western population. High rates of trauma and stigmatization of psychiatric care were noted as possible explanations for this (16). In Yazidi refugees residing in Canada specifically, this has also been noted. For example, Hassan et al. (6) identified that 69.1 percent (56 of 81 Yazidi refugee female patients) who had survived significant trauma, had suspected somatoform conditions. Abdominal and pelvic pain, headaches, dizziness, and hair loss were all acknowledged as part of these suspected somatoform disorders in this population (6). Furthermore, in other countries such as Germany, pain, feelings of suffocation, movement disorders, dizziness and several other somatic symptoms have also been noted amongst Yazidi ISIS survivors as well (17). Therefore, as clinicians, it is imperative that we remain curious, identify, and treat these physical manifestations of mental distress when caring for Yazidis with mental health concerns.

Lastly, the Yazidi women in this study acknowledged that having lost, missing or persecuted family members perpetuates their mental distress. The negative impact of sustained separation of families on mental health in refugee populations post resettlement has been well-established (6, 18, 19; & 20) and should be purposefully acknowledged by health care providers to Yazidi refugees seeking care.

The Yazidi women showed appreciation for Canada’s universal health care and for the specialized refugee clinics which housed their medical care for the first two years upon arrival. However, the preference for seeking support from community, family and loved ones, rather than relying on professional individual encounters, was acknowledged as the favored coping method. This collectivistic approach to mental health has been well established in the literature. Kizilhan (21) explains that “people from traditional-rural regions as a rule are steeped in a collective way of thinking … the individual sees himself as part of a mutually supportive group from which arise the appropriate tasks and obligations” (21). Lobanov-Rostovsky and Kiss (22) also highlight this importance of social connection as a psychosocial intervention in working with displaced female Yazidis. In their review exploring the global evidence on psychosocial interventions for female survivors of conflict related sexual violence, the therapeutic effects of the collective healing power of group interventions were noted (22). However, the barrier of stigma to these group interventions was also emphasized (22). Stigma in group therapeutic interventions was interestingly not something that was emphasized amongst our Yazidi participants but nonetheless should be acknowledged when considering this population’s preference for group interventions.

In summary, psychoeducational interventions tailored to the Yazidi community’s cultural context is imperative. Support for community level interventions that honor this collectivistic method of healing may be more culturally appropriate as opposed to individual therapeutic modalities. Specifically, given the limited exposure to counseling and psychotherapy that many Yazidi refugees would have experienced it is recommended that integrating cultural understandings of illness and healing are key elements to mental health support. This could include recognizing Yazidi beliefs that illness causes are often multifactorial including religious, spiritual or supernatural causes; and focusing on treatment that utilizes protective factors such as ensuring connectedness to each other in their new environments as part of the treatment and move to well-being. As part of this, continued advocacy for family reunification, so that families can heal together, is also critical.

Lastly, it is important to also consider specific cultural and religious factors that may further impact collectivistic (versus individually based) preferred methods of coping. Specifically, the Yazidi traditional three-caste structure is important to acknowledge as a factor that has implications on mental health and help seeking behaviors. Kizilhan (23) details that in this caste system there were Sheikhs (teachers), Pirs (priests) and Murids (lay people). Marriage outside of one’s own case is forbidden as well as was marrying someone from a different religion (23). Furthermore, sexual relations with someone that is not Yazidi traditionally has resulted in exclusion from the Yazidi community (23). Although these strict rules have fortunately been softened for the women coming out of ISIS captivity, this grace has not been extended to the children born out of ISIS captivity. Therefore, historical and transgenerational trauma, along with these social hierarchies, will likely shape preferred coping strategies employed by the Yazidi women. Precisely, these variables will likely influence when women seek out support from their communities, their openness to psychotherapy, or their tendency to isolate and suffer alone.

Several perceived barriers to care were identified. Social determinants of health, such as inadequate housing and social isolation emerged as interconnected factors that perpetuate the post-migration stress Yazidi women face. Several women expressed that their housing is structurally inadequate, often consisting of basement suites that resembled the conditions of their ISIS captivity, triggering traumatic memories. Further, being resettled in neighborhoods far from other Yazidis compounded feelings of loneliness, rendering housing arrangements socially inadequate. Having to work outside of the home, traditionally a male role, and attend school full time (and subsequently not be with their children), were foreign practices that added significant stress. Many Yazidi women described the practice of collectively raising each other’s children as a significant part of their lives back home. These new occupational and educational demands combined with the social isolation they experience post-migration disrupts this communal caregiving, making it difficult to maintain the support systems they relied on for connection. Ultimately, these findings unveil the need to re-think trauma and culturally-informed interventions beyond the clinic. For example, social interventions, such as housing options that are above ground, which can minimize the triggers of captivity, could alleviate some of the trauma. Perhaps providing Yazidi refugees housing in the same complex, where women can collectively support one another may promote the mental health and healing of Yazidi women post-migration.

Language differences, and unrealistic expectations of our Canadian refugee resettlement system were other barriers to care. Being placed in neighborhoods far from other Yazidis, where others do not speak the same language, hinder these women’s chances of building further connection with others here in Canada, and collectively healing as a community. Furthermore, as per management at the refugee clinic, it is our understanding that to receive government funding, attendance at English classes is a requirement of all newcomers (S. Nori, personal communication, September 26^th^, 2024). However, for many of the Yazidi women, this expectation was considered unobtainable. For example, many of these women had never attended traditional schooling and the realities of spending several hours a day travelling to and from school, sitting in a classroom, away from their children, precipitated significant distress.

Resettled refugees’ adherence to the cultural expectations of a host country may evoke at minimum the experience of cultural bereavement. In extreme circumstances, the sentiment of presumed assimilation is also questioned. Authors Tippens et al. (24) emphasize this finding in their work with resettled Yazidi refugee women residing in the midwestern United States. Specifically, they acknowledge Eisenbruch’s (25) model of cultural bereavement, with special reference to the trauma that comes from “the total experience of the uprooted person-or group-resulting from loss of old social structures, cultural values, meanings and self (25, as in 24). Our findings unveil this similar sentiment felt by the Yazidi participants in our study, as they try to navigate these social expectations set for them post-migration. This again raises the question: how do we implement culturally-informed social interventions that avoid perpetuating the distress already experienced by newcomers? Our findings point to the need for creative and culturally-sensitive solutions such as implementing alternative learning environments (for example, flexible outdoor teaching classrooms where grieving families are not separated and children are welcome). Interventions such as this may help to further address these perceived barriers to mental health revealed through our findings.

### Study limitations

Although cultural brokers were present throughout the duration of the focus groups, Yazidi participants may not have felt comfortable speaking freely in front of healthcare providers from a different culture. Furthermore, as the Yazidi community is closely-knit in Calgary, members of the focus group may have inherently known each other. This may have interfered with their ability to speak freely in the group.

## Conclusions

This study is the first known qualitative study exploring Yazidi refugees’ perspectives on their mental health challenges, their preferred coping strategies, and the barriers to receiving mental health care in a Canadian context. Results reveal further insight into potential culturally informed medical and social interventions that can be implemented to improve the mental health care of refugees in Canada in the future.

## Data Availability

The original contributions presented in the study are included in the article/supplementary material. Further inquiries can be directed to the corresponding author/s.
