# Correction: “We cannot live like Canadian”: Yazidi refugees’ perspectives on mental health, coping strategies and barriers to care

**DOI:** 10.3389/fpsyt.2026.1827719

**Published:** 2026-04-16

**Authors:** Jacqueline Bobyn, Bethel Abraham, Nicole Kain, Kimberly Williams, Annalee Coakley, Rita Watterson

**Affiliations:** 1 Department of Psychiatry at University of Calgary, Cumming School of Medicine, Calgary, AB, Canada; 2 Department of Global and Environmental Health, New York University School of Global Public Health, New York, NY, United States; 3 Faculty of Medicine and Dentistry, University of Alberta, Edmonton, AB, Canada; 4 Department of Family Medicine at the University of Calgary, Cumming School of Medicine, Calgary, AB, Canada

**Keywords:** Yazidi, culturally sensitive, refugee mental health, culturally informed care, mental health

The authors Jacqueline Bobyn, Kimberly Williams and Rita Watterson were erroneously assigned to the Department of Family Medicine at the University of Calgary, Cumming School of Medicine, Calgary, AB, Canada. This affiliation has now been removed for these authors and all other affiliations have been renumbered accordingly.

The corrected author list and affiliations read:

“Jacqueline Bobyn^1^, Bethel Abraham^2^, Nicole Kain^3^, Kimberly Williams^1^, Annalee Coakley^4*^ and Rita Watterson^1^

^1^Department of Psychiatry at University of Calgary, Cumming School of Medicine, Calgary, AB, Canada, ^2^Department of Global and Environmental Health, New York University School of Global Public Health, New York, NY, United States, ^3^Faculty of Medicine and Dentistry, University of Alberta, Edmonton, AB, Canada, ^4^Department of Family Medicine at the University of Calgary, Cumming School of Medicine, Calgary, AB, Canada”

An incorrect **Funding** statement was provided. The correct funder is the Geneva Health Forum. The correct **Funding** statement reads:

“The author(s) declare that financial support was received for the research and/or publication of this article. The Article Processing Charges have been partially supported by the Geneva Health Forum. The authors would like to explicitly acknowledge and sincerely appreciate the Geneva Health Forum’s support, which made the open-access publication of this work possible. No additional external funding or grant numbers apply”.

The original version of this article has been updated.

